# Whisky in Tetanus

**Published:** 1877-01

**Authors:** 


WHISKY IN TETANUS.—Dr. Ezra Read, of Terre Haute, Indianna, reports in the *American Practitioner*, a case of traumatic tetanus treated principally with whisky. The case recovered, and the cure was attributed to the daily use of three pints of whisky. Quinine, calomel and chloral were, however, given for five or six days, after which the disease, assuming a somewhat chronic form, continued for a month under the intoxicating doses of whisky. It is not by any means certain that recovery in this case was due to this agent. We remember to have seen a similar case that recovered after several weeks continuance in a kind of chronic form, without the use of whisky. The second and only other case of tetanus we ever saw terminate favorably, was treated with quinine and calomel only.

